# Beta cell antigens in type 1 diabetes: triggers in pathogenesis and therapeutic targets

**DOI:** 10.12688/f1000research.7411.1

**Published:** 2016-04-22

**Authors:** François-Xavier Mauvais, Julien Diana, Peter van Endert

**Affiliations:** 1 Institut National de la Santé et de la Recherche Médical, Unité 1151, Paris, 75015, France; 2 Centre National de la Recherche Scientifique, UMR8253, Paris, 75015, France; 3 Université Paris Descartes, Sorbonne Paris Cité, Paris, 75015, France

**Keywords:** Type 1 Diabetes, Beta Cell Antigen, insulin recognition, Antigen-based immunotherapy, autoreactive T cells

## Abstract

Research focusing on type 1 diabetes (T1D) autoantigens aims to explore our understanding of these beta cell proteins in order to design assays for monitoring the pathogenic autoimmune response, as well as safe and efficient therapies preventing or stopping it. In this review, we will discuss progress made in the last 5 years with respect to mechanistic understanding, diagnostic monitoring, and therapeutic modulation of the autoantigen-specific cellular immune response in T1D. Some technical progress in monitoring tools has been made; however, the potential of recent technologies for highly multiplexed exploration of human cellular immune responses remains to be exploited in T1D research, as it may be the key to the identification of surrogate markers of disease progression that are still wanting. Detailed analysis of autoantigen recognition by T cells suggests an important role of non-conventional antigen presentation and processing in beta cell-directed autoimmunity, but the impact of this in human T1D has been little explored. Finally, therapeutic administration of autoantigens to T1D patients has produced disappointing results. The application of novel modes of autoantigen administration, careful translation of mechanistic understanding obtained in preclinical studies and *in vitro* with human cells, and combination therapies including CD3 antibodies may help to make autoantigen-based immunotherapy for T1D a success story in the future.

## Introduction and context

The key role in type 1 diabetes (T1D) of T lymphocytes recognizing self-antigens expressed by insulin-producing beta cells in pancreatic islets is amply documented and beyond reasonable doubt. As discussed in detail in many excellent reviews (see, for example, 1– 4), a large number of such autoantigens, generally also recognized by autoantibodies, have been identified and are targeted by CD8+ and CD4+ T cells that may contribute to beta cell destruction but can also have a regulatory, protective role. (Pre-)proinsulin ([P]PI), glutamic acid decarboxylase (GAD), the tyrosine phosphatase IA-2, and the zinc transporter ZnT8 play a particularly prominent role and are recognized by autoantibodies detected in routine clinical laboratory assays (see 5, 6 for a discussion of the role of B lymphocytes and autoantibodies in T1D). Given that autoantigens provide specificity to the autoimmune pathology in T1D, major efforts in the scientific field have been devoted, on the one hand, to developing assays for monitoring pathogenic T cell responses against them and, on the other hand, to designing therapeutic strategies specifically silencing such responses.

This short review will take a look at the progress towards the development of diagnostic and therapeutic approaches focusing on T cell autoantigens in the last 5 years. Reviewing the pertinent literature, we will propose three general conclusions. With regard to diagnostic tools, we believe that the number of beta cell proteins and major histocompatibility complex (MHC)-restricted epitopes thereof, as well as the performance of tetramers for the detection of cognate T cells, now provides an increasingly promising basis for monitoring autoreactive T cells. However, as recently argued by Odegard *et al*., assays for antigen-specific cells are still not robust enough for routine use in clinical trials, particularly multicenter trials ^7^. In this context, we will argue that, first, the information obtained in current analyses of such cells is insufficient so that the methods are in need of complementation and, second, that T cell analysis in human T1D should not be limited to self-reactive cells. Concerning autoantigen-based immunotherapy, we agree with Greenbaum and colleagues ^8^ and believe that the general failure of published recent trials calls for more research in preclinical models and *in vitro* with human cells, which should precede small proof-of-concept trials. This being said, occasional discordance between results in the nonobese diabetic (NOD) model and human T1D (e.g. concerning the effect of interleukin [IL]-2 in combination with rapamycin ^9^), and poor reproducibility of some other results in the NOD model ^10^, calls for caution when translating preclinical results to human T1D. Finally, we will argue that important gaps in our understanding of processing, presentation, and T cell recognition of beta cell antigens may have to be addressed to design efficient autoantigen-based immunotherapies.

## T cell epitopes and tetramers

Next to the simple and efficient enzyme-linked immunosorbent spot (ELISpot) assays, tetramers have become standard and increasingly sophisticated tools for detecting both CD8+ and CD4+ autoreactive T cells. Production of these reagents requires identification of MHC-restricted epitopes, the number of which is still increasing. Thus, using mass spectrometric analysis of human leukocyte antigen (HLA) class I eluates ^11^, or prediction algorithm-assisted analysis of PI degradation products produced by the proteasome ^12^, new epitopes presented by four HLA class I alleles, including the disease-associated A*24 and recognized preferentially by patient CD8+ T cells, could be identified. Using the technology of combinatorial labeling of identical tetramers with different sets of multiple fluorochromes, Roep and colleagues developed a tetramer “kit” able to detect T cells specific for multiple dominant autoreactive epitopes simultaneously ^13^. Such kits will be useful where small blood volumes must be analyzed, especially when studying pediatric samples, although even with recent approaches it will be difficult to obtain satisfactory results with blood volumes of significantly less than 20 mL, which realistically can be obtained in trials involving young children. Interestingly, investigators led by von Herrath succeeded in using HLA class I tetramers to analyze pancreatic islets from T1D patients ^14^. Somewhat astonishingly, on average no more than two to nine CD8+ T cells, the dominant lymphocyte type in islet infiltrates, were present per islet; tetramer staining revealed recognition of one or multiple antigens by these cells.

Tetramers have also provided interesting insight into insulin B _9–23_-specific CD4+ T cell responses restricted by the strongly T1D-associated allele HLA-DQ8 (DQB1*03:02). T cells with this specificity were found in six out of 16 patients and recognized denatured but not native antigen. The authors speculate that the disulfide bridges in native insulin might inhibit processing by myeloid cells, perhaps suggesting special processing pathways overcoming this inhibition in pancreatic islets (see also below) ^15^. DQ8-restricted CD4+ T cells recognizing PI (C peptide in this case) were also found in islet infiltrates of a T1D patient and among blood lymphocytes of several T1D patients ^16^. Interestingly, in both studies, autoreactive DQ8-restricted T cells were exclusively found in patients, an unusual feature given that autoimmunity generally leads to amplification and activation but not *de novo* appearance of autoreactive cells, which are also present in healthy individuals. As recently reviewed by Ehlers and Rigby ^17^, it is well documented that self-reactive T cells tend to have a naïve phenotype in healthy individuals but a memory phenotype in subjects with T1D.

ZnT8 has also joined the ranks of CD4+ T cell-recognized autoantigens in human T1D. T1D patients responded to a larger number of ZnT8 epitopes with greater proliferative responses ^18^; moreover, ZnT8-specific CD4+ T cells were skewed towards T helper 1 (Th1) cells in T1D patients, while Th2 and IL-10-producing cells were prevalent in healthy adults ^19^. We have found that ZnT8 is a major autoantigen for CD8+ T cells in pediatric diabetes ^20^; as transfer of ZnT8-specific human CD8+ T cells can induce diabetes in HLA-humanized mice, such cells may play an important role in the human pathology ^21^.

Because tetramer detection of human CD4+ T cells recognizing islet cell antigens generally requires their prior expansion, methods for quantitative assessment of such cells in untouched lymphocytes could be of substantial interest. Eugster and colleagues analyzed sequences of 1650 T cell receptors (TCRs), obtained from six patients, recognizing the dominant DR4-restricted epitope GAD 557I and then used next-generation sequencing to determine the frequency of individual TCR sequences among patient CD4+ cells, which was found to rank from <0.00001 to 1.6% ^22^. Disappointingly, there were almost as many different TCR sequences as T cell clones, suggesting that specific TCR-targeted therapies have little chance of success.

## Novel insights on frequency and expression signatures of self-reactive T cells

While the vast majority of research efforts concerning autoreactive T cells has focused on detecting and enumerating such cells, possibly combined with limited functional analyses, recent studies suggest that exploration of adaptive autoimmunity should go beyond this step and aim to perform in-depth characterization of phenotypic and functional properties. One critical finding is the abundance of the self-reactive T cell repertoire in healthy individuals and the surprisingly limited purging of it during thymic education. Yu and colleagues found that T cells recognizing male antigens were only threefold more abundant in females than in males, with full cytotoxic potential and equivalent tetramer staining intensity for cells from male and female donors. Moreover, the frequency range of self-reactive cells was similar to that of cells recognizing foreign antigens ^23^. As an example, the frequency of T cells recognizing a dominant insulin epitope (B _10–18_) was 1 in 10 ^4^, with only a 2.66-fold increase in T1D patients. Similar findings were reported by Maeda *et al*. for T cells recognizing Melan-A ^24^. However, in both studies, self-reactive CD8+ T cells were anergic when challenged with antigen, reflected in distinct gene expression profiles including low Bcl-2 expression and up-regulation of CTLA-4. Interestingly, anergic self-reactive CD8+ T cells may not only indicate the absence of auto-aggressive responses in healthy individuals but also play a beneficial role in patients with ongoing autoimmunity, since such cells increase in frequency upon treatment with CD3 antibodies ^25^.

Another study suggested that not only is looking at gene expression profiles of self-reactive cells more informative than counting cells but also screening for self-reactivity can sometimes be omitted when searching for gene expression profiles with prognostic value in organ-specific autoimmune diseases. McKinney and associates compared gene expression by single CD8+ T cells from patients with chronic infections and patients with autoimmune diseases and found a profile indicative of T cell exhaustion with good clinical outcome in the latter but poor outcome and response to therapy in the former setting ^26^. A single surrogate marker, the anti-apoptotic transcription factor KAT2B, correlated with progression to disease in children at risk of T1D and with triggering of autoimmunity in the NOD model. Thus, one might say that one of the most interesting surrogate markers of progression to T1D described so far was identified while ignoring the antigenic specificity of the CD8+ T cells examined. Why a surprisingly large number of peripheral blood T cells would express a signature indicating progressive disease is unclear; however, this may be related to systemic immune and metabolic alterations in pre-diabetic children, such as a type 1 interferon (IFN) transcription signature ^27^ and altered lipid and amino acid metabolism ^28^. Whatever the reason for the findings of McKinney *et al*., they indicate that the search for surrogate markers of disease progression or immunotherapeutic effects in T1D should not be limited to self-reactive T cells or, to be more precise, should not be limited to cells detectable with tetramers. This is not only because systemic effects of a type 1 IFN signature and of metabolic perturbation might produce surrogate markers in non-autoreactive cells but also because many autoreactive cells are likely to escape detection due to the frequently low affinity of self-reactive T cells. This was impressively demonstrated by Sabatino and colleagues, who used a novel method (micropipette adhesion frequency assay) to show that low-affinity, tetramer-undetectable but fully functionally competent T cells outnumbered tetramer-detectable cells in both an anti-viral and an autoimmune response ^29^. In conclusion, recent technological advances, in particular single cell technology, have opened up new avenues for the identification of surrogate markers of T1D risk, rate of progression, and response to immunotherapy. These technologies should now be applied to the analysis of different immune cell populations from T1D patients including, but not limited to, self-reactive T lymphocytes.

## Are self-reactive T cell responses unique?

Results obtained in the last 5 years have provided more evidence for some unique features preferentially found in self-reactive T cell responses. One of them is the unusual interaction among TCR, peptide, and MHC molecule resulting in weak avidity. Lamont and colleagues identified a self-peptide (an epitope in the insulin A chain) lacking the C-terminal anchor residue and thus extending the list of self-epitopes filling MHC peptide-binding sites only partially, a feature thought to facilitate escape from thymic negative selection ^30^. Bulek and colleagues found another mechanism resulting in “ultra-weak” TCR-pMHC interaction (studying presentation of PPI _15-24_ by HLA-A2), in which an extremely peptide-centric TCR-pMHC interaction limited TCR-MHC interactions to a “light touch” unlikely to be sufficient for negative selection ^31^. Post-translational epitope modification in the periphery (in this case by transglutaminase) is another mechanism allowing epitopes to escape negative selection and has recently been suggested to be implicated in the autoantigenicity of a chromogranin A epitope (one of the epitopes filling the peptide binding site partially) recognized by the popular BDC2.5 CD4+ T cells ^32^. Similarly, citrullination of the GRP78 protein specifically in the NOD beta cells induces the translocation of this chaperone from the endoplasmic reticulum to the plasma membrane and generates a novel autoantigen recognized by effector T cells ^33^.

An additional mechanism contributing to escape from thymic deletion is the phenomenon of type B CD4+ T cells. This type of T cell, identified first and studied in depth by Unanue’s group, recognizes cognate peptides exogenously added to antigen-presenting cells (APCs), but not peptides produced within APCs from native antigen (at least when peptide loading occurs in standard H-2M-equipped intracellular compartments) ^34^. In the NOD model, Mohan and colleagues found that the majority of islet-infiltrating CD4+ T cells recognizing insulin B _9–23_ were of type B. Having previously shown that type B clones transferred diabetes, the group demonstrated that a mouse expressing the transgenic type B TCR 8F10 on a RAG knockout background developed T1D with faster kinetics than an analogous mouse expressing a standard type A TCR (recognizing both exogenous and APC-processed peptide) ^35^. Interestingly, diabetes was also triggered by 8F10 cells in mice lacking pancreatic lymph nodes, suggesting that stimulation of type B T cells can occur directly in islets, bypassing for unknown reasons the requirement for priming by APCs migrating to lymph nodes documented for other T cells recognizing islet antigens. Presumably, dendritic cells (DCs) reaching into islet vessels attract such cells ^36^; whether direct expression of MHC class II by beta cells, recently confirmed to occur in the NOD model, plays a role in recruiting type B T cells, or any autoreactive CD4+ T cells, into islets remains to be determined ^37^.

## Insulin recognition by CD4+ T cells

Insulin is arguably the “number one” autoantigen in the NOD model of T1D, not only because of its expression restricted to beta cells, the association of its genetic polymorphism with disease risk, and the role of its recognition in initial triggering of the autoimmune response ^38^ but also because the structural basis of its recognition by CD4+ T cells is well understood and provides more evidence for the key role of unusual TCR-pMHC interactions in T1D. The insulin B chain fragment 9–23 has long been known as the key self-epitope recognized by murine and human CD4+ T cells. Examining the interaction of this peptide with the murine MHC class II molecule I-A ^g7^, Stadinski and colleagues identified four possible “binding registers” of which number 1 and 2 were most efficient ^39^. Surprisingly, four different T cell clones examined recognized register 3, the least efficient register due to a conflict between the p9 peptide residue and the p9 binding site pocket. The authors proposed that particularly poor autoantigen presentation underlies the predisposing effect of some MHC class II alleles in T1D and postulated that only the very high insulin concentrations in islets allow for the formation of MHC complexes with poor ligands, consistent with a model proposed by Unanue and colleagues ^40^. Given that activation of many T cells recognizing register 3 can be ameliorated by removing a glutamic acid in p8 (residue B _21_), the authors speculated that an islet-specific processing (i.e. proteolytic) event might remove this residue, thus creating a “cryptic” epitope absent during thymic T cell selection ^41^.

A similar study by Unanue’s group agreed that I-A ^g7^ can bind B _9–23_ in multiple registers; however, the authors did not find any T cells recognizing register 3 ^42^. Standard type A T cells recognized the efficient register 1, but type B cells dominating responses upon immunization with B _9–23_ reacted with register 2 presented poorly by I-A ^g7^.

Because I-A ^g7^–restricted CD4+ T cell responses to B _10–23_ trigger islet cell autoimmunity, studying specific responses in non-autoimmune mice expressing I-A ^g7^ might reveal insight into the regulatory mechanisms that protect against T1D. Pauken and colleagues found specific T cells both in NOD mice and in C57BL/6 mice expressing I-A ^g7^ ^43^. Interestingly, in the pancreatic lymph nodes of young NOD mice, i.e. in early pre-diabetes, the majority of these cells were anergic or expressed Foxp3 and IFN-γ expression was limited to intra-islet cells. However, in non-autoimmune B6/I-A ^g7^ mice, B _10–23_ reactive cells remained naïve, suggesting that they did not encounter antigen. Therefore, islet insults or inflammation in the NOD mouse are likely to precede priming of key autoreactive CD4+ T cells.

Collectively, the molecular and functional characterization of autoreactive T cells from patients and in the NOD model raises a number of intriguing questions. How common is “ultra-weak” TCR/pHLA interaction in human T1D, and what are the mechanisms underlying low-avidity interactions? Can the micropipette adhesion assay reveal an additional autoreactive repertoire in human T1D? Are type B CD4+ T cells as critical in humans as they are in the NOD model? Finally, are there specific processing/proteolytic events producing “cryptic” epitopes in pancreatic islets?

## Antigen-based immunotherapy trials in patients

Given encouraging results of prior preclinical or human pilot studies, hopes had been raised that antigen-based immunotherapy might provide a safe and efficient strategy to prevent or even cure human T1D. Unfortunately, the trials concluded in recent years have almost completely failed to provide any evidence of clinical efficacy. Treatment starting with nasally applied insulin at T1D onset of 52 adult patients not requiring exogenous insulin had no effect on C peptide levels or progression to dependence on exogenous insulin ^44^, thereby repeating a previous failure of a trial of oral insulin. Immunization of 12 patients with newly diagnosed T1D with insulin B chain in incomplete Freund’s adjuvant induced robust insulin-specific adaptive responses, but a clinical effect was not noted ^45^. Two independent and relatively large trials in which patients were injected within 3 months of disease onset with GAD and alum adjuvant also showed no effect on C peptide levels or insulin requirement ^46, 47^. However, as has been commented on by others ^48^, the regimens used in the GAD trials differed in numerous parameters (dose, route of injection, timing relative to disease onset, and use of alum adjuvant) from those tested in preclinical experiments, making trial failure appear less surprising. The single glimpse of hope published derives from a trial in which a PI-encoding plasmid was injected for 12 weeks after disease onset ^49^. One of the regimens resulted in a transient increase of C peptide levels by 15% (vs. -10% for placebo) at 15 weeks after start of treatment. The authors also noted a decrease in CD8+ T cells specific for one peptide/HLA combination, although it was not clear whether this decrease was related to the transient clinical response.

Globally, it is safe to say that antigen-based therapy is far from having met expectations and that new avenues must be explored to produce more encouraging results. Presently, immunomodulatory approaches, particularly CD3 antibodies, remain the “gold standard” with respect to efficacy ^50, 51^, although transient EBV reactivation by anti-CD3 dosing schemes with the best clinical efficacy has been a source of concern ^52^. Only short-term treatment with CD3 antibodies has been able to suppress the rise in insulin requirement over a period of 48 months ^53^. Others better placed than we are have made recommendations on how to make progress, which we wish to reiterate here ^8, 48, 54^. The need for better biomarkers has been formulated repeatedly but remains as urgent as ever. More research on the natural history of T1D, including the issues formulated above, is required to design more efficient therapies. *In vitro* studies with human samples, and large-scale in-depth characterization of the autoimmune response as outlined above, should be performed. The focus should be on preventing T1D onset. Small pilot studies based on rigorous hypothesis testing in the preclinical model and including mechanistic outcome studies should be preferred to large studies as performed in the past. Novel therapeutic strategies developed in the NOD model may also point the way to more efficient antigen-based immune intervention.

## Novel strategies for antigen-based immune therapy of T1D

The last few years have seen a wealth of novel ideas on how to treat or prevent T1D, most of them developed and tested in the NOD model. One of these uses targeting of complete autoantigens to specific APC receptors to induce regulatory responses. Targeting of self or non-self proteins to the DC asialoglycoprotein receptor induces suppressive T regulatory type 1 (Tr1) cells secreting large amounts of IL-10 but little tumor necrosis factor-α, IFN-γ, and IL-2, which are all produced upon targeting of the same proteins to Dectin-1, DC-SIGN, or Lox1 ^55^. Interestingly, the approach also works in cynomolgus macaques; however, its efficacy in a pre-existing autoimmune setting has not been tested. Another approach uses *Lactococcus lactis* engineered to secrete PI and IL-10; in combination with low-dose parenteral anti-CD3, oral administration of these bacteria to freshly diabetic mice can induce lasting remission of T1D, possibly due to the induction of PI-specific regulatory T cells found in the intestinal mucosa and in islets ^56^.

Various other recent strategies use self-epitopes to attenuate the autoimmune response. Injection of 10-week-old mice with a lentivirus, driving exclusive expression of B _9–23_ in hepatocytes due to a microRNA sequence, induces peptide-specific CD8+ T cells and regulatory T cells and halts the progression of T1D; combining the virus with low-dose anti-CD3 may induce remission of T1D ^57^. Administration of a super-agonist variant of B _9–23_ with strongly enhanced stimulatory capacity provides complete protection from T1D if this peptide is delivered in subimmunogenic doses via an osmotic pump, starting at a young age, or via a DEC205-targeted fusion antibody. The protective effect was reflected in an increase of Foxp3+ T cells and a decrease in IFN-γ and IL-17 production. However, the necessity of administration during very early pre-diabetes and of determining the precise subimmunogenic dose will complicate the practical application of this strategy ^58^. Others have proposed using TCR-like antibodies blocking or deleting specific autoreactive T cell populations ^59, 60^, or to produce tetramers targeting presumably pathogenic T cells and eliminating them by inducing apoptosis or by directly killing them through tetramer conjugation with saponin ^61, 62^. Some of these approaches can reduce the number of the targeted T cells significantly *in vivo* and delay or reduce onset of T1D. Determining biomarker profiles of patients prior to antigen-based immunotherapy may be key for the success of such treatments.

Probably the most exciting novel approach was reported during the drafting of this article by the group of Santamaria ^63^. This group had previously demonstrated protection from spontaneous diabetes in the NOD model upon stimulation and expansion of autoantigen-experienced CD8+ T cells by nanoparticles coated with cognate MHC-class I/peptide complexes ^64^. Applying this approach to stimulating CD4+ T cells by nanoparticles coated with MHC class II/peptide complexes, the group could now show that the novel strategy can not only prevent and revert T1D in the NOD model but also attenuate autoimmune inflammation in rodent models of collagen-induced autoimmune arthritis and in experimental autoimmune encephalomyelitis. Remarkably, the approach even works when T cells recognizing subdominant epitopes are targeted. Applied to patients, this would obviate the need for identifying dominant or triggering epitopes and T cell populations, thereby removing one of the major obstacles to epitope-based immunotherapy in the outbred human population. The strategy seems to work by converting Th1 memory cells to Tr1 cells producing IL-10 and transforming growth factor-β, which in turn induce the differentiation of cognate regulatory B lymphocytes producing IL-10. Expanded Tr1 and regulatory B lymphocytes then suppress autoimmunity in a synergistic manner, presumably both by direct effects on effector T cells and by suppressing the pro-inflammatory action of cognate APCs ^63^. A limitation of the strategy is the need to maintain bi-weekly treatment, since about half of NOD mice relapse with disease upon interruption of treatment.

## Concluding remarks

The period reviewed in this article has witnessed significant progress in the mechanistic understanding of autoantigen recognition and the development of some novel tools for monitoring the cellular autoimmune response in T1D. However, much remains to be learned with respect to the phenotype and function of pathogenic and protective human T cells, and surrogate markers of disease progression and response to therapy are still urgently needed. Moreover, the initial events activating autoreactive T cells and subsequent factors, both genetic and environmental, resulting in expansion rather than silencing of such cells remain poorly understood. The very recent finding that islet-infiltrating CD4+ T cells both in the NOD model and in T1D patients can recognize covalently linked hybrids of PI peptides with other beta cell peptides highlights the need to better understand the peculiar antigen processing environment in the islet organ ^65^. This important finding may also suggest that the antigens used in previous trials of antigen-specific immunotherapy may not have been optimal. We anticipate that the application of recent technologies to highly multiplexed analysis of gene and protein expression by single human cells will make an important contribution to identifying surrogate markers and advancing our mechanistic understanding of human T1D. Recent results also suggest that simple oral or parenteral administration of autoantigens may stand little chance in achieving remission of human T1D. Given that CD3 antibodies so far have produced the most promising results, combining antigen administration with anti-CD3 has become a preferred option for many in the field. The future will show whether novel approaches such as those cited above can obviate the need for broadly immunomodulatory components in combination therapies or enhance their therapeutic efficacy.

## Abbreviations

APC, antigen-presenting cell; DC, dendritic cell; ELISpot, enzyme-linked immunosorbent spot; GAD, glutamic acid decarboxylase; HLA, human leukocyte antigen; IFN, interferon; IL, interleukin; MHC, major histocompatibility complex; NOD, nonobese diabetic; (P)PI, (pre-)proinsulin; T1D, type 1 diabetes; TCR, T cell receptor; Tr1, T regulatory type 1.
